# The Relationship between Smoking Habits and Serum Levels of 8-OHdG, Oxidized LDL Antibodies, Mn-SOD and Carotenoids in Rural Japanese Residents

**DOI:** 10.2188/jea.13.29

**Published:** 2007-11-30

**Authors:** Koji Suzuki, Yoshinori Ito, Junichi Ochiai, Kunio Aoki, Kenji Wakai, Akiko Tamakoshi, Masahiko Ando, Yoshiyuki Watanabe, Kotaro Ozasa, Nao Seki, Yoshikazu Nishino, Takaaki Kondo, Yoshiyuki Ohno

**Affiliations:** 1 Department of Public Health, Fujita Health University School of Health Sciences.; 2 Aichi Cancer Center.; 3 Department of Preventive Medicine/Biostatistics and Medical Decision Making, Nagoya University Graduate School of Medicine.; 4 Department of Social Medicine and Cultural Sciences, Research Institute for Neurological Diseases and Geriatrics, Kyoto Prefectural University of Medicine.; 5 Division of Public Health, Department of Infectious Disease Control and International Medicine, Niigata University Graduate School of Medical and Dental Science.; 6 Division of Epidemiology, Department of Public Health and Forensic Medicine, Tohoku University Graduate School of Medicine.; 7 Department of Public Health/Health Information Dynamics, Nagoya University Graduate School of Medicine.

**Keywords:** smoking, 8-OHdG, oxidized LDL antibody, Mn-SOD, carotenoids

## Abstract

This study investigated the relationship between smoking habits and serum levels of 8-OHdG, oxidized LDL antibodies (oLAB), Mn-SOD, and carotenoids. Subjects were 79 males (mean age ± standard deviation; 62.1 ± 10.0 years) and 79 females (60.3 ± 10.3 y) who attended a health examination screening in the town of Hokkaido, Japan. Serum 8-OHdG, Mn-SOD, and oLAB levels were measured by ELISA and serum carotenoids levels were measured by HPLC. Smoking habits were assessed by public health nurses using a questionnaire. Serum 8-OHdG levels were significantly higher in males than in females. On the other hand, serum levels of *β*-carotene, *α*-carotene, *β*-cryptoxanthin, and zeaxanthin and lutein were significantly lower in males than in females. Serum *β*-carotene, *β*-cryptoxanthin, and zeaxanthin and lutein were significantly lower in males who were current smokers, compared to non-smokers. Serum 8-OHdG levels were also significantly higher in current smokers. Furthermore, in males, serum oLAB and *β*-carotene levels were significantly and negatively correlated with the number of cigarettes smoked per day. Serum Mn-SOD levels were unrelated to smoking habits in males. In conclusion, this preliminary study suggests that cigarette smoking increases serum 8-OHdG levels and reduces serum levels of oLAB and carotenoids, such as *β*-carotene, *β*-cryptoxanthin, and zeaxanthin and lutein in healthy male subjects. Serum levels of 8-OHdG, oLAB, and carotenoids may be useful biomarkers of oxidative conditions affected by smoking.

Smoking is known to generate reactive oxygen species (ROS) in vivo.^1^ It has been reported that ROS, such as superoxide radical, singlet oxygen, and hydroxyl radical, are involved in aging, mutagenesis, and carcinogenesis.^2^^,^^3^ There are a variety of enzymatic and non-enzymatic systems that defend membranes and DNA against free radicals in vivo.

Carotenoids, such as *β*-carotene, may protect cells from oxidative stress by quenching free radicals.^4^ Fruits and vegetables are rich in carotenoids and serum carotenoids levels are higher in people who eat greater amounts of these foods.^4^ It has also been reported that serum carotenoids levels are lower in smokers than in non-smokers.^5^^,^^6^ Some follow-up studies^7^^-^^9^ have shown that incidences of certain types of cancer, such as lung, stomach, and colon cancer, are lower in subjects with high serum *β*-carotene levels.

Superoxide dismutase (SOD) is one of antioxidant enzymes that defend against superoxide anions and protect the cell against oxidation.^10^ There are three different forms of SOD that have been found in mammalian cells: copper and zinc SOD, manganese SOD (Mn-SOD), and extracellular SOD.^10^^-^^12^ Mn-SOD is located in the mitochondrial matrix and plays an important role in dismutation of the superoxide anions in tissues.^11^ It has been reported that serum Mn-SOD levels are higher in patients with primary hepatoma, gastric cancer, and acute myocardial infarction (MI),^13^ but few previous studies have reported the relationship between smoking habits and serum Mn-SOD levels.

Oxidized low-density lipoprotein (oLDL) is generated by the action of ROS and is believed to play a critical role in the development and progression of atherosclerosis.^14^ Oxidized LDL is taken up by macrophages, and oLDL antibodies (oLAB) are present both in atherosclerotic lesions and plasma.^15^ In some studies,^16^^-^^18^ serum oLAB levels have been related to the degree of atherosclerosis.

The deoxyribonucleic acid (DNA) adduct 8-hydroxydeoxyguanosine (8-OHdG) is one of the important products generated by ROS from deoxyguanosine. It produces predominantly transversion mutations (G to T).^19^ 8-OHdG is one of the biomarkers for oxidative DNA damage^20^ and the levels of 8-OHdG in DNA and urine are higher in subjects with malignant neoplasms.^21^ There has been no reported investigation of the relationship between smoking and these oxidants and antioxidants based on different localization and substantial origins in the same subjects.

We have studied the relationship between serum levels of oxidants or antioxidants and mortality from lung cancer in a large-scale cohort study. First, we performed a preliminary study that investigated the validity of their measurement methods and the cancer-caused biological components as significant biomarkers in large-scale specimens. In this study, we investigated the relationship between these biological factors and smoking habits that are strongly associated with lung cancer.

## METHODS

We randomly selected 158 subjects (79 males and 79 females) by sex from 882 inhabitants who attended a health examination screening in an area of Hokkaido in August 2001. This is one of the participating areas of the Japan Collaborative Cohort (JACC) Study.^22^ The response rate for the health examination was about 90% of applicant for this examination. All subjects were 40 to 74 years old and worked primarily in fishing, dairy farming, or commerce.

Trained public health nurses administered a questionnaire regarding health and daily lifestyle habits. We assessed smoking status (current smoker, ex-smoker, or non-smoker) and the number of cigarettes smoked per day at the time of examination.

Fasting blood samples were taken during the examination and within one hour sera were separated from blood cells by centrifugation. These samples were stored in a deep freezer at −80°C until they were analyzed in September 2001.

We obtained written informed consent from the participants for providing information and serum to our epidemiological study.

Serum 8-OHdG levels were measured by enzyme-linked immunosorbent assay (ELISA) method (8-OHdG Check high sensitivity; Japan Institute for the Control of Aging, Fukuroi, Japan). A microtiter plate was coated with 8-OHdG. The lowest 8-OHdG standard (0.125 ng/mL) was further diluted 1:1 (0.0625 ng/mL) and 1:3 (0.0313 ng/mL) with phosphoric acid buffer because serum 8-OHdG levels were lower than 0.125 ng/mL in most of the subjects. Therefore, we tried to set the standard range from 0.0313 to 10 ng/mL. Fifty microliters of the filtration sample and standard were added to each well. Fifty microliters of anti-8-OHdG antibody was added to all wells and the plate was incubated at 4°C overnight. After the wells were washed four times, 100 *μ*L of peroxidase-conjugated secondary antibody was added to all wells and the plate was incubated at room temperature (RT) for 60 minutes. After the wells were washed four times, 100 *μ*L of substrate containing tetramethylbenzidine was added to all wells and the plate was incubated at RT in the dark for 12 minutes. One hundred microliters of stop solution containing phosphoric acid was added to all wells and the absorbance was read with an ELISA microwell reader at 450 nm.

Serum oLAB levels were measured by ELISA method (oLAB ELISA kit; Biomedica, Wien, Austria). Serum Mn-SOD levels were measured by ELISA method (Mn-SOD ELISA SYSTEM; Amersham Biosciences Corp., Piscataway, New Jersey, USA). They were measured according to the attached instruction manuals. The measurement times of these analyses were about three hours. For the intra-assay (n = 8) and inter-assay (n = 5) reproducibility, the coefficients of variation (CVs) for oLAB and Mn-SOD were found to be less than 10% (Table 1).

**Table 1.** The intra- or inter- assay CVs for ELISA kits of serum 8-OHdG, oLAB and Mn-SOD

Item	mean value^a^	intra-assay	inter-assay
8-OHdG(ng/ml)	0.19 ± 0.08	22.7%	
	0.18 ± 0.08		35.7%
oLAB(mU/ml)	731 ± 26	3.5%	
	297 ± 28		9.3%
Mn-SOD(ng/ml)	269 ± 22	8.4%	
	271 ± 27		9.2%

a: mean value ± standard deviation

Serum levels of *α*- and *β*-carotenes, lycopene, *β*-cryptoxanthin, and zeaxanthin and lutein were measured separately by high-performance liquid chromatography (HPLC).^23^ The CVs of intra- and inter-assay were less than 10%.^23^

Serum levels of total cholesterol and *γ*-GTP activity were measured by auto-analyzer (JCA-RX20, Nihon Denshi Co., Ltd.).

All statistical analyses such as *t* test, analysis of variance (ANOVA), and chi-square test were conducted using a statistical package (Stat View^®^, Version 5.0, SAS). Since serum levels of 8-OHdG, oLAB, and carotenoids had logarithmic distributions, the *t* test, calculation of simple and partial correlation coefficients, and ANOVA were conducted using log-transformed values of these components. Partial correlations between serum 8-OHdG, oLAB, and Mn-SOD levels and the number of cigarettes smoked per day in males were adjusted for age and alcohol consumption. Correlations between serum carotenoids levels and the number of cigarettes smoked per day were adjusted for confounding factors, i.e., age, alcohol consumption, BMI, serum total cholesterol levels, and *γ*-GTP activity. These adjusting factors were considered to strongly affect serum carotenoids levels. To compare serum levels of 8-OHdG, oLAB, Mn-SOD, and carotenoids by smoking habits, ANOVA with Bonferroni tests were used. In females, the number of current smokers was too small to conduct these analyses in relation to the number of cigarettes smoked.

Serum levels of 8-OHdG, oLAB, and carotenoids were expressed as a median, with a range of 25 to 75%, and serum Mn-SOD levels were expressed as a mean value ± standard deviation.

## RESULTS

Table 1 showed the intra- and inter-assay reproducibility of the ELISA kit. For the intra-assay reproducibility (n = 8), the CV for 8-OHdG was found to be over 20%, excluding 8-OHdG. For the inter-assay reproducibility (n = 5), the CVs for 8-OHdG were over 30%.

Table 2 compared lsvels of serum 8-OHdG, oLAB, Mn-SOD, and carotenoids between males and females. The proportions of current smokers and drinkers were significantly higher in males than in females. Serum 8-OHdG levels were significantly higher in males than in females, but there were no significant differences in serum Mn-SOD and oLAB levels. Serum levels of *β*-carotene, *α*-carotene, *β*-cryptoxanthin, and zeaxanthin and lutein were significantly higher in females than in males.

**Table 2.** Comparison of serum levels of 8-OHdG, oLAB, Mn-SOD, and carotenoids by sex.

Item	Males	Females	p
n	79	79	

Age(y)	62.1 ± 10.0	60.3 ± 10.3	0.993^a^

Current smoker(%)	40.6	10.2	0.001>^b^

Current alcohol drinker(%)	51.9	11.9	0.001>^b^

8-OHdG(ng/mL)	0.14 (0.05-0.39)	0.07 (0.03-0.20)	0.002^a^

oLAB(mU/mL)	287 (175-457)	295 (184-508)	0.434^a^

Mn-SOD(ng/mL)	175 ± 44	166 ± 45	0.125^a^

*β*-Carotene(*μ*mol/L)	0.449 (0.253-0.729)	0.863 (0.526-1.381)	0.001>^a^

*α*-Carotene(*μ*mol/L)	0.093 (0.064-0.176)	0.157 (0.099-0.227)	0.001>^a^

Lycopene(*μ*mol/L)	0.307 (0.184-0.440)	0.307 (0.205-0.461)	0.377^a^

*β*-Cryptoxanthin(*μ*mol/L)	0.167 (0.103-0.259)	0.242 (0.183-0.353)	0.001>^a^

Zeaxanthin&Lutein(*μ*mol/L)	1.012 (0.722-1.257)	1.191 (0.862-1.572)	0.009^a^

Data excluding age and Mn-SOD are represented as median with 25-75% range in parentheses.

Age and Mn-SOD is represented as mean values ± standard deviation.

a: t test, b: chi-square test

Table 3 showed serum levels of 8-OHdG, oLAB, Mn-SOD, and carotenoids according to smoking habits in males. Serum 8-OHdG levels were significantly higher in current smokers than in non-smokers. There were no significant differences in serum levels of oLAB and Mn-SOD by smoking habits. Serum levels of *β*-carotene, *β*-cryptoxanthin, and zeaxanthin and lutein were significantly lower in current smokers than in non-smokers. Furthermore, serum levels of *β*-cryptoxanthin and zeaxanthin and lutein were significantly lower in ex-smokers than in non-smokers. Serum *α*-carotene levels tended to be lower in current smokers than in non-smokers, but there was no significant difference in serum lycopene level. When current smokers were divided into two groups, light (1-20 cigarettes/day) and heavy (>20 cigarettes/day), there was a significant and inverse dose-response relationship between serum levels of carotenoids, excluding lycopene, and the number of cigarettes smoked per day. Serum oLAB levels tended to be lower in heavy smokers than in non-smokers.

**Table 3.** Comparison of serum levels of 8-OHdG, oLAB, Mn-SOD, and carotenoids by smoking status in males.

Item		non-smokers	ex-smokers	current smokers	p ^a^	current smokers divided by cigarettes/day		p ^b^
						1-20	>20	
n		21	30	28		14	14	

8-OHdG	(ng/mL)	0.07	0.14	0.30 *	0.043			
		(0.03-0.15)	(0.05-0.29)	(0.07-0.50)		0.32	0.19	0.098
						(0.10-0.52)	(0.03-0.49)	
oLAB	(mU/mL)	291	352	214	0.166			
		(194-452)	(220-538)	(137-350)		259	177	0.195
						(134-563)	(107-343)	
Mn-SOD	(ng/mL)	173 ± 64	171 ± 32	181 ± 39	0.119			
						183 ± 44	180 ± 36	0.237

*β*-Carotene	(*μ*mol/L)	0.729	0.480	0.359	0.001>			
		(0.430-1.174)	(0.244-0.743)	(0.194-0.483)		0.372 *	0.219 **	0.001>
						(0.259-0.501)	(0.158-0.397)	
*α*-Carotene	(*μ*mol/L)	0.144	0.093	0.086	0.070			
		(0.077-0.236)	(0.064-0.179)	(0.055-0.112)		0.086	0.085 *	0.054
						(0.057-0.148)	(0.043-0.103)	
Lycopene	(*μ*mol/L)	0.328	0.241	0.318	0.169			
		(0.287-0.656)	(0.172-0.413)	(0.123-0.520)		0.384	0.205	0.208
						(0.190-0.458)	(0.108-0.543)	
*β*-Cryptoxanthin	(*μ*mol/L)	0.259	0.148 *	0.147 **	0.009			
		(0.166-0.386)	(0.093-0.244)	(0.100-0.192)		0.157	0.103 *	0.015
						(0.117-0.226)	(0.063-0.173)	
Zeaxanthin&Lutein	(*μ*mol/L)	1.184	0.915 **	0.983 **	0.001			
		(1.044-2.012)	(0.690-1.099)	(0.718-1.309)		0.983	0.759 *	0.003
						(0.791-1.393)	(0.501-1.310)	

Data excluding Mn-SOD are represented as median with 25-75% range in parentheses.

Mn-SOD is represented as mean values ± standard deviation.

a: p value by ANOVA of three groups (non-smokers, ex-smokers, and current smokers)

b: p value by ANOVA of four groups (non-smokers, ex-smokers, current smokers smoked 1-20 cigarettes/day, and current smoker smoked >20 cigarettes/day )

*: p <0.05, **: p<0.01 (Bonferroni test compared to non-smokers)

Table 4 showed the correlation coefficients between the number of cigarettes smoked per day and serum levels of 8-OHdG, oLAB, Mn-SOD, and carotenoids. Serum levels of oLAB and *β*-carotene showed significant and inverse correlations with the number of cigarettes smoked per day after adjusting for confounding factors. Serum 8-OHdG levels tended to be positively correlated with the number of cigarettes smoked per day when adjusted for confounding factors. In male smokers, serum oLAB levels were inversely correlated with the number of cigarettes smoked per day, but there was no significant relation between serum levels of 8-OHdG, Mn-SOD, or carotenoids and the number of cigarettes smoked per day.

**Table 4.** Correlation between serum oxidant or antioxidant levels ^a^ and the number of cigarettes smoked per day in males.

Item	Simple correlation coefficients	Partial correlation ^b^ coefficients	Partial correlation ^b^ coeffecients (current smokers)
8-OHdG	0.198 $	0.200 $	0.140
oLAB	-0.260 *	-0.263 *	-0.322 $
Mn-SOD	0.127	0.058	-0.081

*β*-Carotene	-0.330 **	-0.277 *	-0.106
*α*-Carotene	-0.221 $	-0.182	-0.234
Lycopene	-0.060	-0.131	0.052
*β*-Cryptoxanthin	-0.175	-0.118	0.078
Zeaxanthin&Lutein	-0.099	-0.035	0.042

a: Correlation coefficients were calculated using log-transformed serum oxidant or antioxidant levels excluding Mn-SOD and the number of cigarettes smoked per day

b: Partial correlation between serum 8-OHdG, oLAB and Mn-SOD and the number of cigarettes smoked per day adjusted for age, alcohol consumption. Partial correlation between serum carotenoids and the number of cigarettes smoked per day adjusted for age, BMI, alcohol consumption, serum total cholesterol and *γ*-GTP activity

$: p <0.1, *: p <0.05, **: p <0.01

In males, serum 8-OHdG levels showed a significant inverse correlation with oLAB (r = −0.292, *p* = 0.011 [simple correlation coefficient]). Furthermore, serum oLAB levels were not related to serum total cholesterol levels in either sex (males: r = 0.020, *p* = 0.985; females: r = 0.142, *p* = 0.213).

## DISCUSSION

Cigarette smoke contains about 3800 chemicals including ROS.^1^ DNA damage induced by ROS is quickly repaired in vivo by exonucleases and isolated 8-OHdG is excreted in urine without further metabolism.^24^ The levels of 8-OHdG have been studied as a biomarker for oxidative DNA damage.^19^^-^^21^ Urinary 8-OHdG levels are known to be elevated in patients with malignancies^21^ and 8-OHdG levels in DNA are reported to be higher in cancerous tissues than in noncancerous lesions.^25^^,^^26^

In this study, serum 8-OHdG levels were significantly higher in males than in females. In males, serum 8-OHdG levels were higher in current smokers than in non-smokers, but they were not related to the number of cigarettes smoked per day after adjustment for confounding factors.

There are few studies that have investigated the relation between serum 8-OHdG levels and sex or smoking habits. It has been reported that urinary 8-OHdG levels are higher in smokers than in non-smokers,^27^ and this is in agreement with the relationship we found in this study. On the other hand, it has been reported that there is no difference in 8-OHdG levels in DNA between males and females.^28^ The level of 8-OHdG in peripheral leukocytes has been reported to be significantly higher at 10 minutes after smoking, compared to before smoking, whereas the level was only slightly higher in smokers compared to non-smokers.^1^ Those results confirm the existence of an efficient DNA repair mechanism for the rapid removal of 8-OHdG from leukocytes and may result in no difference in DNA 8-OHdG levels between males and females.^28^

Our study suggests that higher serum 8-OHdG levels reflect a higher smoking rate for males. In non-smokers, there was no significant difference in serum 8-OHdG levels between males and females in this study (median: males, 0.07 ng/mL; females, 0.06 ng/mL, *p* = 0.883).

In this study, intra- and inter-assay reproducibility of the ELISA test for serum 8-OHdG is not good (>20%). Although 8-OHdG in plasma can be measured by HPLC,^29^ the method is complicated and is not available for epidemiological studies. Furthermore, the measured value by HPLC (mean value for adult controls: 13pg/mL [range: 4.3-21.2 pg/mL]^29^) is lower than that by ELISA. The reason for that difference is unclear, but it was suggested that HPLC can detect only free 8-OHdG, whereas ELISA can detect total 8-OHdG, including 8-OHdG in oligonucleotide.^30^ The method to measure serum 8-OHdG levels should be improved, so its levels could be a useful biomarker for oxidation in vivo.

The oLDL is believed to play a role in the progression of atherosclerosis.^14^ Oxidized LDL accumulates in macrophages, which turn into foam cells and form atherosclerotic plaques. This mechanism stimulates the immune system and oLAB is produced.

It has been reported that there is no significant difference in serum oLAB levels between males and females^31^ and we obtained the same results. Serum oLAB levels tended to be lower in current smokers than in non-smokers and were significantly and negatively correlated with the number of cigarettes smoked per day in males.

ROS causes an increase of oLDL, and oLDL reacts with oLAB. But serum oLAB levels were negatively related to smoking, which normally generates ROS. Serum oLAB levels are reported to be negatively correlated with plasma oLDL levels in healthy subjects^31^ due to the possible role of oLAB in maintaining the low level of oLDL. From these findings, it was suggested that serum oLAB levels were lower in healthy subjects with oxidative stress. Most of our study subjects were healthy. Therefore, it may be reasonable that serum oLAB levels showed a negative correlation with the number of cigarettes smoked per day and with serum 8-OHdG levels. In an experimental study,^32^ chronic exposure to cigarette smoke was reported to inhibit antibody response. Serum oLAB levels might be related to the generation of ROS and individual immunoresponse, including the inhibition of antibody response.

Mn-SOD is localized in the mitochondrial matrix and plays a role in dismutation of superoxide anion in tissues.^13^ It has been reported that Mn-SOD synthesis is induced by cytokines that play a role in inflammatory reactions, such as interleukin-1 and tumor necrosis factor.^33^^,^^34^

In this study, there was no significant difference in serum Mn-SOD levels between males and females. Serum Mn-SOD levels were slightly and insignificantly higher in current smokers compared to non-smokers, and they were unrelated to smoking dose.

Previous studies also reported that there was no significant difference in serum Mn-SOD levels between males and females.^13^ Serum Mn-SOD levels have been reported to be higher in patients with cancer and acute MI.^13^ Our study suggests that serum Mn-SOD is released from damaged tissues in patients with cancer and MI. There may be no significant difference, however, in serum Mn-SOD levels by sex or smoking habits in subjects without serious diseases.

Carotenoids, such as *β*-carotene and zeaxanthin, also have antioxidant effects and play an important role in defending against ROS.^35^ In fact, it has been reported that serum levels of carotenoids such as *β*-carotene, zeaxanthin, and *β*-cryptoxan-thin are lower in smokers than in non-smokers.^5^^,^^6^ We obtained the same results in this study. Furthermore, serum *β*-carotene levels were significantly and negatively correlated with the number of cigarettes smoked per day. But in male smokers, there was no significant correlation between carotenoids and the number of cigarettes smoked per day. It has been reported that there is an inverse dose-response relationship between serum *β*-carotene levels and the number of cigarettes smoked per day.^36^ It has also been reported that the number of cigarettes smoked per day is unrelated to serum carotenoids levels in moderate and heavy smokers.^37^ Fifty percent of male smokers in our study smoked more than 20 cigarettes per day. Thus, it is not surprising that there was no correlation between serum carotenoids levels and the number of cigarettes smoked per day among current smokers. Serum carotenoids, especially *β*-carotene, might be useful biomarkers of oxidative stress caused by smoking in vivo. Some studies have reported that there is no effect of smoking on serum lycopene levels^5^^,^^38^ and we obtained same results.

It has been reported that serum carotenoids levels are positively correlated with the frequency of consumption of vegetables or fruits among the subjects in this same area.^39^ Moreover, the intake of vitamin C, vitamin E, *β*-carotene, and fiber among smokers has been reported to be lower compared with that of non-smokers^40^ In this study, the difference in serum levels of carotenoids, such as *β*-carotene, *β*-cryptoxanthin, and zeaxanthin and lutein, between current smokers and non-smokers was clear, compared to other oxidant or antoxidant levels. We suggest that this clear relationship between smoking habits and serum carotenoids levels may be affected not only by oxidative stress, such as smoking, but also by the difference in nutrient intake. On the other hand, there have been few reports that have investigated the relationship between serum levels of 8-OHdG, oLAB, and Mn-SOD and food intake frequencies.

This preliminary study demonstrated that smokers had higher serum levels of serum 8-OHdG and lower serum levels of oLAB and carotenoids, such as *β*-carotene, *β*-cryptoxanthin, and zeaxanthin and lutein, compared to non-smokers. Serum levels of 8-OHdG, oLAB, and carotenoids were useful biomarkers of an oxidative condition affected by smoking in vivo. Serum Mn-SOD levels were slightly higher in current smokers compared to non-smokers, but further studies are needed.

In conclusion, serum levels of 8-OHdG, oLAB, and carotenoids, such as *β*-carotene, *β*-cryptoxanthin, and zeaxanthin and lutein, were associated with smoking habits, but serum levels of Mn-SOD and lycopene were not. These results suggest that serum levels of 8-OHdG, oLAB, and carotenoids may be useful biomarkers for smoking-related diseases, such as lung cancer.

## APPENDIX

The present members of the JACC Study and their affiliations are as follows: Dr. Akiko Tamakoshi (present chairman of the study group), Nagoya University Graduate School of Medicine; Dr. Mitsuru Mori, Sapporo Medical University School of Medicine; Dr. Yutaka Motohashi, Akita University School of Medicine; Dr. Ichiro Tsuji, Tohoku University Graduate School of Medicine; Dr. Yosikazu Nakamura, Jichi Medical School; Dr. Hiroyasu Iso, Institute of Community Medicine, University of Tsukuba; Dr. Haruo Mikami, Chiba Cancer Center; Dr. Shuji Hashimoto, Fujita Health University School of Medicine; Dr. Yutaka Inaba, Juntendo University School of Medicine; Dr. Yoshiharu Hoshiyama, Showa University School of Medicine; Dr. Hiroshi Suzuki, Niigata University School of Medicine; Dr. Hiroyuki Shimizu, Gifu University School of Medicine; Dr. Hideaki Toyoshima, Nagoya University Graduate School of Medicine; Dr. Shinkan Tokudome, Nagoya City University Graduate School of Medicine; Dr. Yoshinori Ito, Fujita Health University School of Health Sciences; Dr. Shogo Kikuchi, Aichi Medical University School of Medicine; Dr. Akio Koizumi, Graduate School of Medicine and Faculty of Medicine, Kyoto University; Dr. Takashi Kawamura, Kyoto University Center for Student Health; Dr. Yoshiyuki Watanabe, Kyoto Prefectural University of Medicine, Research Institute for Neurological Diseases and Geriatrics; Dr. Tsuneharu Miki, Kyoto Prefectural University of Medicine; Dr. Chigusa Date, Faculty of Human Environmental Sciences, Mukogawa Women’s University ; Dr. Kiyomi Sakata, Wakayama Medical University; Dr. Takayuki Nose, Tottori University Faculty of Medicine; Dr. Norihiko Hayakawa, Research Institute for Radiation Biology and Medicine, Hiroshima University; Dr. Takesumi Yoshimura, Institute of Industrial Ecological Sciences, University of Occupational and Environmental Health, Japan; Dr. Katsuhiro Fukuda, Kurume University School of Medicine; Dr. Naoyuki Okamoto, Kanagawa Cancer Center; Dr. Hideo Shio, Shiga Medical Center; Dr. Yoshiyuki Ohno, Nagoya University Graduate School of Medicine; Dr. Tomoyuki Kitagawa, Cancer Institute of the Japanese Foundation for Cancer Research; Dr. Toshio Kuroki, Gifu University; and Dr. Kazuo Tajima, Aichi Cancer Center Research Institute.

The past investigators of the study group were listed in the reference^26^^)^ except for the following seven members (affiliations are those where they participated in the study): Dr. Takashi Shimamoto, Institute of Community Medicine, University of Tsukuba; Dr. Heizo Tanaka, Medical Research Institute, Tokyo Medical and Dental University; Dr. Shigeru Hisamichi, Tohoku University Graduate School of Medicine; Dr. Masahiro Nakao, Kyoto Prefectural University of Medicine; Dr. Takaichiro Suzuki, Research Institute, Osaka Medical Center for Cancer and Cardiovascular Diseases; Dr. Tsutomu Hashimoto, Wakayama Medical University; and Dr. Teruo Ishibashi, Asama General Hospital.
